# Tidal levels significantly change bacterial community composition in a tropical estuary during the dry season

**DOI:** 10.1007/s42995-024-00254-w

**Published:** 2024-10-08

**Authors:** Pablo Aguilar, Chantima Piyapong, Nitcha Chamroensaksri, Pachoenchoke Jintasaeranee, Ruben Sommaruga

**Affiliations:** 1 Department of Ecology, University of Innsbruck, Innsbruck, Austria; 2 Microbial Complexity Laboratory, Instituto Antofagasta and Centre for Bioengineering and Biotechnology (CeBiB), University of Antofagasta, Antofagasta, Chile; 3 Department of Biotechnology, Faculty of Marine Sciences and Biological Resources, University of Antofagasta, Antofagasta, Chile; 4 Millennium Nucleus of Austral Invasive Salmonids-INVASAL, Concepción, Chile; 5 Department of Biology, Faculty of Science, Burapha University, Chonburi, 20131 Thailand; 6 Center of Excellence On Environmental Health and Toxicology (EHT), OPS, Ministry of Higher Education, Science, Research and Innovation (MHESI), Bangkok, 10400 Thailand; 7 National Biobank of Thailand (NBT), National Center for Genetic Engineering and Biotechnology (BIOTEC), National Science and Technology Development Agency (NSTDA), Pathum Thani, 12120 Thailand; 8 Department of Aquatic Science, Faculty of Science, Burapha University, Chonburi, 20131 Thailand

**Keywords:** Rivers, Brackish systems, Tides, Bacterial indicators, Water pollution

## Abstract

Estuaries are usually characterized by strong spatial and temporal variability in water physicochemical conditions and are often largely affected by human activities. One important source of variability is caused by tides that can swiftly alter not only physicochemical conditions but also the abundance and composition of the biota. The effect of the diurnal tidal cycle on microbial community composition during different seasons remains uncertain, although this knowledge underlies having effective monitoring programs for water quality and potential identification of health risk conditions. In this study, we assessed the bacterioplankton community composition and diversity across four tidal water levels in a tropical estuary characterized by a mixed semidiurnal tide regime (i.e., two high and two low tides of varying amplitudes) during both dry and wet seasons. The bacterial community composition varied significantly among the four tidal levels, but only during the dry season, when the influence of the seawater intrusion was largest. Bacterial indicators’ taxa identified using the Indicator Value Index were found within Cyanobacteria, Actinobacteriota, Bacteroidota, and Proteobacteria. The indicator taxon *Cyanobium* sp. had a prominent presence across multiple tidal levels. The main predicted phenotypes of the bacterial communities were associated with potential pathogenicity, gram-negative, and biofilm formation traits. While there were no marked predicted phenotypic differences between seasons, pathogenic and gram-negative traits were more prevalent in the dry season, while biofilm formation traits dominated in the wet season. Overall, our findings underscore the intricate relationship between river hydrodynamics and bacterial composition variability and hint a significant human impact on the water quality of the Bangpakong River.

**Supplementary Information:**

The online version contains supplementary material available at 10.1007/s42995-024-00254-w.

## Introduction

Rivers discharging on the coast contribute to the biogeochemical cycles of surrounding ecosystems, the global water balance, the sediment input to the ocean, and climate variability (Moftakhari et al. 2013; O’Connor et al. 2022; Raymond et al. 2013). They are complex ecosystems where tides, water discharge, and seasonality converge to produce highly dynamic environmental conditions (Hoitink and Jay 2016). In regions where rivers are influenced by tides, the ocean influences up to hundreds of kilometers landward (Hoitink and Jay 2016). The area of the river affected by tides is referred to as an estuary, which is divided into lower, middle, and upper zones (Elliott and McLusky 2002). The boundaries dividing these zones are not fixed and undergo continual shifts in response to changes in river discharge (Day et al. 2012).

Each coastal environment typically exhibits a specific type of tidal regime. According to Swinbanks (1982), tides can manifest in three main patterns during each lunar day, namely diurnal, mixed-semidiurnal, and semidiurnal. The first pattern refers to one high and one low tide, the second refers to two high and two low tides of different ranges, and the third one consists of two high and two low tides of approximately equal size (De Boer et al. 1989). These patterns influence many hydrological and physical properties along the discharge channel. For example, they modulate river discharge volumes (Ensign et al. 2013; Sassi and Hoitink 2013) and affect environmental factors such as nutrient concentration, salinity, and temperature, that in turn affect, for example, phytoplankton biomass (Lee et al. 2018; Tao et al. 2020). These changes also affect the diversity, abundance, and metabolic activity of microorganisms (Chen et al. 2019b; Guizien et al. 2014; Jovanovic et al. 2017). For example, the spring-neap tidal dynamics, which refer to the variations in tidal range occurring due to the alignment of the sun and moon, are known to influence the abundance and composition of microorganisms in upper, middle, and lower estuaries (Chen et al. 2019a).

The microbial community of rivers is largely affected by climate, local environmental conditions, and hydrological properties (Clark et al. 2022; URycki et al. 2020). Notably, headwater regions often have a higher microbial diversity than the river mouth (Savio et al. 2015). Anthropogenic disturbances along the river can also affect the microbial community structure (Battin et al. 2016; Findlay 2010; Pusch et al. 1998). Thus, microbes are a good proxy for detecting the contamination of different sources. For example, indicator taxa such as *Escherichia coli* and enterococci have been the indicators of water quality degradation for years (Murray et al. 2001). While most of the past research on water quality monitoring has focused on individual bacterial taxa, the potential of assessing the entire microbial communities is currently recognized (Astudillo‑García et al. 2019). In this context, molecular and bioinformatic tools have resulted in the use of novel indicators (McLellan and Eren 2014), such as the Indicator Value Index (IndVal) that represents a robust method to detect deterioration in the quality of freshwater systems by identifying a combination of microbial indicators. This tool, for example, has pinpointed distinct indicator taxa associated with alterations in oxygen concentrations (Spietz et al. 2015), as well as with diverse estuary types and zones (Alonso et al. 2022).

In the present study, we addressed the question as to whether changes in bacterial community composition occur within the diurnal tide dynamics and whether these changes are season dependent. Although recent studies have assessed the relationship between tidal cycles and the structure of aquatic microbial communities, they have focused on temperate and subtropical estuaries (Gong et al. 2024). Therefore, the aim of our study was to assess the composition and diversity of the bacterial community in the upper section of a tropical estuary, which is characterized by a mixed semidiurnal tidal regime and low water quality. We hypothesized that the seasonal variation and diurnal tidal cycles significantly affect bacterial diversity and phenotypic traits, with the dry season exhibiting more pronounced changes due to increased sediment resuspension and microbial community shifts at lower tidal levels. We have not only tested the tidal effects on these parameters but also identified potential bacterial indicators of poor water quality. Both the dry and wet seasons were considered because we expected to identify the most significant shift in community composition, when water discharge decreases, and the effect of ocean intrusions intensifies during the dry season. Accordingly, water samples were collected in both seasons during four distinct tidal stages—higher high water (HHW), lower high water (LHW), higher low water (HLW), and lower low water (LLW) in the Bangpakong River, Thailand.

## Materials and methods

### Sampling site

The Bangpakong River is one of the most important lotic systems flowing into the inner Gulf of Thailand. The tide is mixed-semidiurnal and the tidal range at the river mouth is mesotidal (< 3.3 m). The river provides water for urban areas, household consumption, industry use, and agricultural irrigation, which have resulted in the release of contaminants into the river (Ma and Chanpiwat 2022). However, there are natural factors such as seawater intrusion, riverbank erosion and flooding that also influence the water quality of the river (Ma and Chanpiwat 2022). The river watershed is located between 13°05′–14°30′ N and 100°57–102°00′ E with a catchment of 19,786 km^2^ (Boonphakdee et al. 1999) and reaching 240 km in length from the headwater in the forest hilly ranges to the river mouth (Okwala et al. 2020).

The area is significantly affected by the humid Southeastern monsoon from the end of May until the middle of November, as well as the arid Northeastern monsoon from late November to February. Consequently, the climatic pattern is bifurcated into two distinct periods: a rainy season extending from June through November and a dry spell lasting from December until May. The average monthly flow of freshwater undergoes seasonal fluctuations, peaking in September with an average of 1300 m^3^/s and dropping to its lowest in February with an average of 10 m^3^/s (Boonphakdee et al. 1999). The salt intrusion can travel 150 km upstream where there is a conjunction of two tributaries that merge into the main Bangpakong River (Bordalo et al. 2001). The average depth along the river channel is 8 m, but the shallow area of the river estuary is 0.5–2.5 m deep and located near the river mouth (Boonphakdee et al. 2008). The precise location for water sampling was at site BK-03, known as Wat Hua Sai situated at 13°46′39.4″ N, 101°12′10.3″ E (Supplementary Fig. 3).

### Sampling strategy

The Bangpakong River flows through the Chachoengsao Province and has a total length of 122 km (Ma and Chanpiwat 2022). Sample collection was done in the middle of the river (with depths between 7 and 10 m) at the upper estuary of the Bangpakong River, as depicted in Supplementary Fig. 3.

The river cross-section at the sampling site was measured using a portable depth sounder (HONDEX PS-7FL). Water column profiles (temperature and salinity) were measured with a CTD Profiler (Model SD208). Furthermore, a JFE Advantech (Infinity AEM-USB) device measured water velocity and flow direction at mid-water depth, recording readings every 15–36 min during each sampling session, depending on tidal characteristics. In addition, physicochemical parameters (water temperature, salinity, pH, dissolved oxygen, and total dissolved solids) were measured in situ with a multi-parameter probe (YSI 556).

Water samples were collected during the dry season in February 2020 and the wet season in September 2020, specifically during equivalent spring tides. During these seasons, the sampling was meticulously conducted to cover an entire tidal sequence, specifically through the points of LLW, HLW, LHW, and HHW. At each tidal level, three independent replicates at the surface (< 0.3 m depth) were collected with a 5-L water sampler.

### DNA extraction and sequencing

One liter of each water replicate was directly filtered through a 0.22-μm pore filter (Supor^®^, Pall Corporation) using a peristaltic pump without prefiltration. Filters were removed from the filter unit with sterile scalpels and preserved in sterile tubes (15 mL) at − 80 °C until subsequent DNA extraction at the National Biobank of Thailand (NBT) and the National Science and Technology Development Agency (NSTDA), Thailand. DNA was extracted from each sample using ZymoBIOMICS™ DNA Miniprep Kit (Zymo Research, D4300). The filtered membrane filter was dissected and placed into the ZR BashingBead™ lysis tube (Zymo Research Corporation), and then, DNA integrity was checked by agarose gel electrophoresis and quantified in a NanoDrop^TM^1000 spectrophotometer (Thermo Scientific, DE, USA). A minimum concentration of 10 ng/µL was used for sequencing in the Illumina MiSeq platform.

DNA was used as a template for the amplification of the V3–V4 region of the 16S SSU rRNA using the Quick-16S Primer Set V3–V4 (Zymo Research, Irvine, CA). The primers used include 341f (CCTACGGGDGGCWGCAG, CCTAYGGGGYGCWGCAG) and 806r (GACTACNVGGGTMTCTAATCC; Caporaso et al. 2011). The forward primer 341f is a mixture of the two sequences listed, based on the original sequence described by Herlemann et al. 2011. Sequencing was done at ZymoBIOMICS (Irvine, CA). Briefly, the preparation of the sequencing library utilized a cutting-edge method for setting up libraries. Real-time PCR equipment was employed for conducting PCR assays to manage cycles effectively and minimize the creation of PCR chimeras. Subsequent PCR outcomes were measured using qPCR fluorescence indicators, and then amalgamated at uniform molarity levels. This consolidated library underwent a purification process using the Select-a-Size DNA Clean & Concentrator kit (supplied by Zymo Research, located in Irvine, CA). Quantification followed, utilizing both TapeStation (from Agilent Technologies in Santa Clara, CA) and Qubit (provided by Thermo Fisher Scientific in Waltham, WA). Sequencing of the final library was executed on the Illumina MiSeq platform, incorporating a v3 reagent kit capable of 600 cycles, with a 10% infusion of PhiX. As a standard reference, the ZymoBIOMICS Microbial Community DNA Standard (sourced from Zymo Research in Irvine, CA) was integrated as a positive control during each specific library preparation phase. To monitor potential contamination levels introduced during the laboratory procedures, negative controls, comprising both a sterile extraction control and a library preparation control, were systematically incorporated. Raw amplicon reads were deposited in NCBI’s Sequence Read Archive (SRA) under Accession PRJNA977079.

### Amplicon data processing

The analysis of raw amplicons from 24 samples was conducted utilizing the DADA2 R package, version 1.16.0 (Callahan et al. 2016). Upon the examination of the quality profiles of the reads, adjustments were made, cutting forward reads down to 265 bases and trimming reverse reads to 215 bases. Any reads presenting more than two expected errors were excluded from the process. Learning from a subset consisting of 933,503 reads helped ascertain the error rates, which subsequently assisted in deducing the ASVs. The procedure combined forward and reverse reads, facilitating the acquisition of the comprehensive denoised ASV sequences. Any sequences exhibiting one or more discrepancies in the overlapping section were discarded. The “removeBimeraDenovo” function was employed for the elimination of chimeras. Finally, ASV taxonomical classification was done using the Silva reference dataset version 138 throughout the IDTAXA algorithm (Murali et al. 2018). This approach led to the creation of a table enumerating read counts and the taxonomic details for all identified ASVs. The filtering process excluded samples with fewer than 10,000 sequences and those sequences identified as eukaryotes, archaea, chloroplasts, mitochondria, or undetermined. For data normalization, the study applied a variance-stabilizing transformation (vds/vst) within the R environment, utilizing the DESeq2 package (Love et al. 2014).

### Amplicon analyses

Alpha diversity metrics (ASV number, Shannon, and inverse Simpson) and the phylogenetic diversity (Faith’s PD) were calculated using the Vegan and Picante packages within R environment, respectively (Fine and Kembel 2011; Oksanen et al. 2019; R Core Team 2018). The phylogenetic tree used for Faith’s PD (Faith 1992) was calculated using FastTree v. 2.1.7, applying the generalized time-reversible model. A Kruskal–Wallis test was performed to test for significance between tidal levels (low vs. high) and between seasons (dry vs. wet). The analysis of multivariate homogeneity of group dispersion (betadisper), ordinations (metaMDS), fit of environmental vectors into ordinations, and statistical differences among tidal levels (ANOSIM) were calculated in the Vegan package in R (Oksanen et al. 2019). A permutation analysis (*n* = 999) was done in betadisper to test for significance using the permutest function in the same package.

Identification of taxonomic members that varied within and across seasons was achieved through a linear discriminant analysis of effect size (LEfSe) utilizing the Galaxy platform (http://huttenhower.sph.harvard.edu/galaxy/) (Segata et al. 2011). Initially, an analysis employing the Kruskal–Wallis test (with an alpha of 0.05) was performed to examine the distinctive distribution of values across various categories. Subsequently, a Wilcoxon test conducted on a pairwise basis (also with an alpha of 0.05) ensured that comparisons between all subcategories within different overarching classes were in concordance at the class level.

Finally, the results were used to build a linear discriminant analysis model (threshold = 2.0) that was crucial for discerning and ranking these unique taxonomic constituents based on their variance between and within the seasonal shifts.

The algorithm BugBase (Ward et al. 2017) was used to predict nine phenotype categories: gram staining, oxygen tolerance, ability to form biofilms, mobile element content, pathogenicity, and oxidative stress tolerance. Briefly, BugBase integrates information from multiple sources, including the Integrated Microbial Genomes (IMG; Markowitz et al. 2014), the Kyoto Encyclopedia of Genes and Genomes (KEGG) through PICRUSt (Langille et al. 2013), and the Pathosystems Resource Integration Center (PATRIC; Snyder et al. 2007). This comprehensive approach helps in pinpointing how particular operational taxonomic units (OTUs) contribute to phenotypic traits at the community level. By harnessing data across these platforms, BugBase facilitates a nuanced understanding of the microbial community’s functional capabilities. The web-based interface of BugBase, accessible at http://bugbase.cs.umn.edu, was employed to extract phenotypic data derived from 16S rRNA gene sequences. To ascertain variations among water levels and across different seasons, a Mann–Whitney–Wilcoxon test was applied with an alpha level of 0.05. This method provided a statistical framework for identifying significant disparities in the datasets, highlighting phenotypic differences based on environmental and temporal conditions.

To differentiate bacterial indicator groups across various tidal levels, we ran an indicator value analysis (IndVal) using the indicspecies package within the R environment (Cáceres and Legendre 2009). This analysis was based on relative abundance data. The IndVal method identifies species combinations that display significant specificity (component A) and fidelity (component B) to particular tidal levels. In brief, to forecast the combinations of amplicon sequence variants (ASVs), involving up to three ASVs (to keep computational times reduced), we used the “indicator”" function with the top 500 most prevalent ASVs. Later, to select the combination with the highest IndVal, we used the “pruneindicators” function while ensuring a fidelity exceeding 80% (component B).

## Results

### Physicochemical parameters

**Fig. 1:**
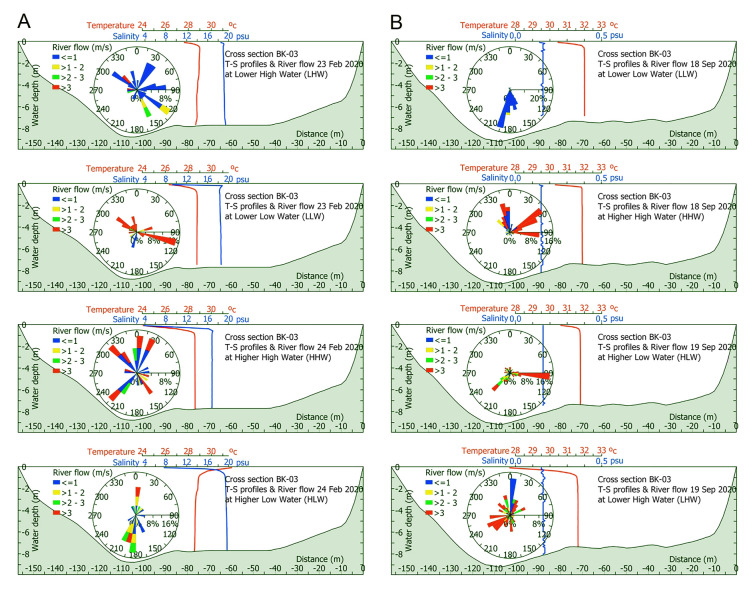
River cross-section at the BK-03 sampling site depicting water column temperature, salinity, river depth, and width during the dry (**A**) and wet (**B**) seasons at different tidal levels. Current speed and direction are indicated by a rose diagram

In general, large differences in the height of the tidal levels between seasons were not observed (Supplementary Fig. 1). The only noticeable difference was observed in the higher low water level (HLW), which was 1.8 m and 1.3 m during the dry and wet seasons, respectively. In the dry season, the river flow velocity was highly variable within and among tidal levels (CV = 55–179%), with LLW mainly having a rate > 3 m/s and LHW mostly having lower values (< 1 m/s) (Fig. 1). The flow velocity also showed a high variability (CV = 64.7–77.8%) during the wet season and the mean value increased following the rising tide (LLW = 0.29 m/s, HLW = 3 m/s, LHW = 3.4 m/s, and HHW = 3.7 m/s). The direction of the river flow (0° = north and 180° = south, to the river mouth) was more variable in the dry season than that in the wet one. In the latter season, and at the LLW level, the flow was mainly southward (153°—200°; i.e., direction to the river mouth), whereas at the HHW level, it was northward and driven by the tidal influence (313°—97°).

The water temperature was slightly higher during the wet season (mean temperature, dry = 28.7 °C and wet = 31.8 °C), but variation was virtually absent among tidal levels in both seasons (CV_dry_ = 0.6%, CV_wet_ = 0.7%). The highest salinity values were measured at HLW (mean salinity: 19.3) and LHW (mean salinity: 19.1), followed by LLW (mean salinity: 18.3) and HHW (mean salinity: 16.5) with a coefficient of variation of 7% among tides during the dry season. Instead, the salinity varied minimally during the wet season (mean salinity: 0.200 ± 0.004, CV = 2.6%).

Dissolved oxygen and total dissolved solids (Supplementary Table 1) were both higher in the dry season (mean DO: 6.14 ± 0.2 mg/L; mean TDS: 19.1 ± 1 g/L) than those in the wet one (mean DO: 3.00 ± 0.06 mg/L; mean TDS: 0.3 ± 0.005 g/L). Instead, pH values remained similar in both seasons (dry = 7.4; wet = 7.0).

### Bacterial community diversity and composition

**Fig. 2:**
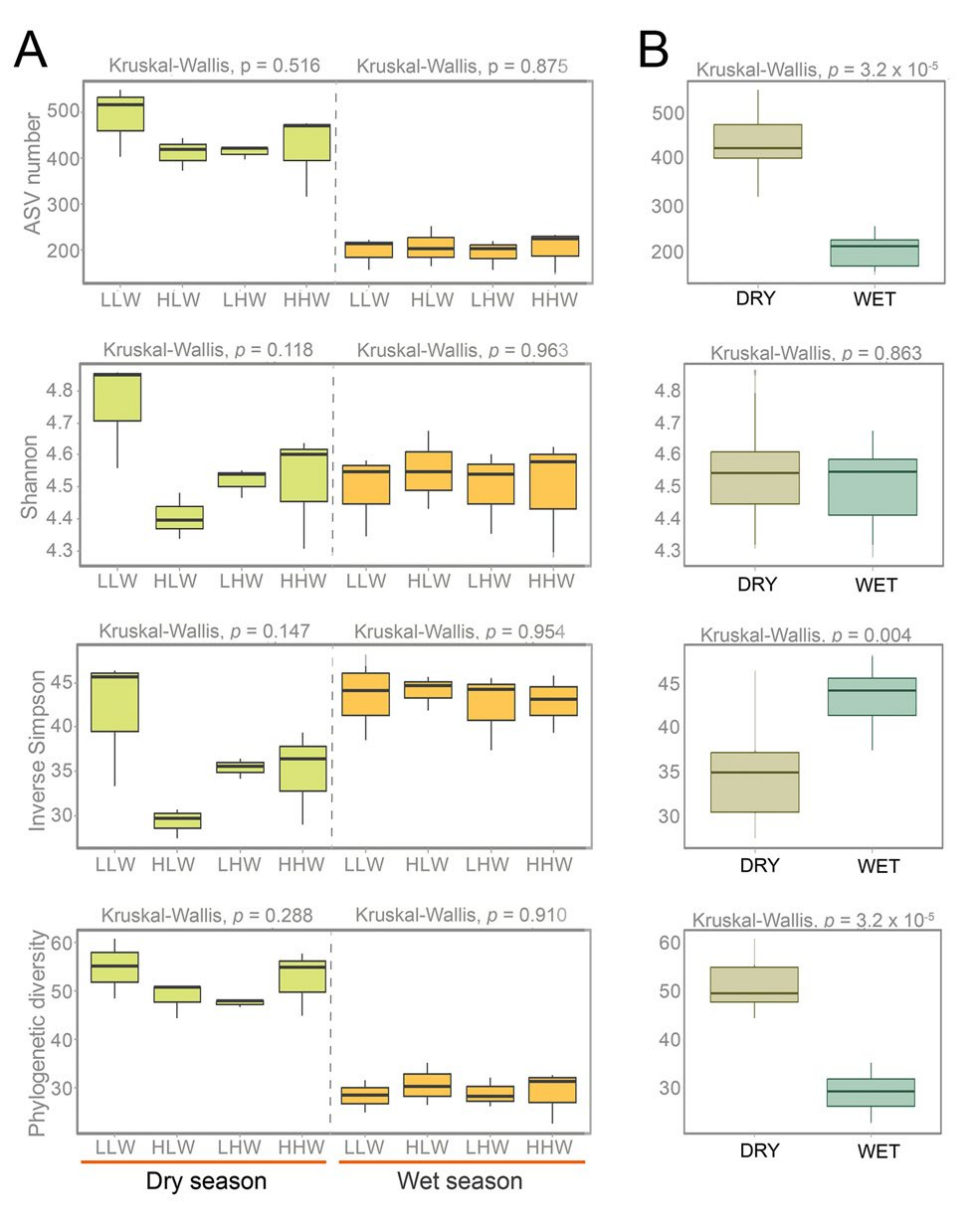
Estimates of ASV number, Shannon diversity, inverse Simpson diversity, and phylogenetic diversity (Faith’s PD) for the bacterial community from the different tidal levels (**A**). Diversity metrics are also compared between seasons (**B**). *HHW* higher high water, *LHW* lower low water, *HLW* higher low water, *LLW* lower low water

Alpha diversity metrics for the different tidal levels were more variable in the dry season than those in the wet season (Fig. 2A). Significant differences were observed only when comparing diversity metrics between seasons (Fig. 2B). For example, the number of ASVs and phylogenetic diversity were significantly higher in the dry season, whereas the Inverse Simpson index was significantly higher during the wet one (Kruskal–Wallis test, *p* = 0.004). No statistical differences were found among the different tidal levels (Kruskal–Wallis test, *p* > 0.05). This was also true when the low (LLW and HLW) and high (LHW and HHW) tides were compared within each season.

**Fig. 3:**
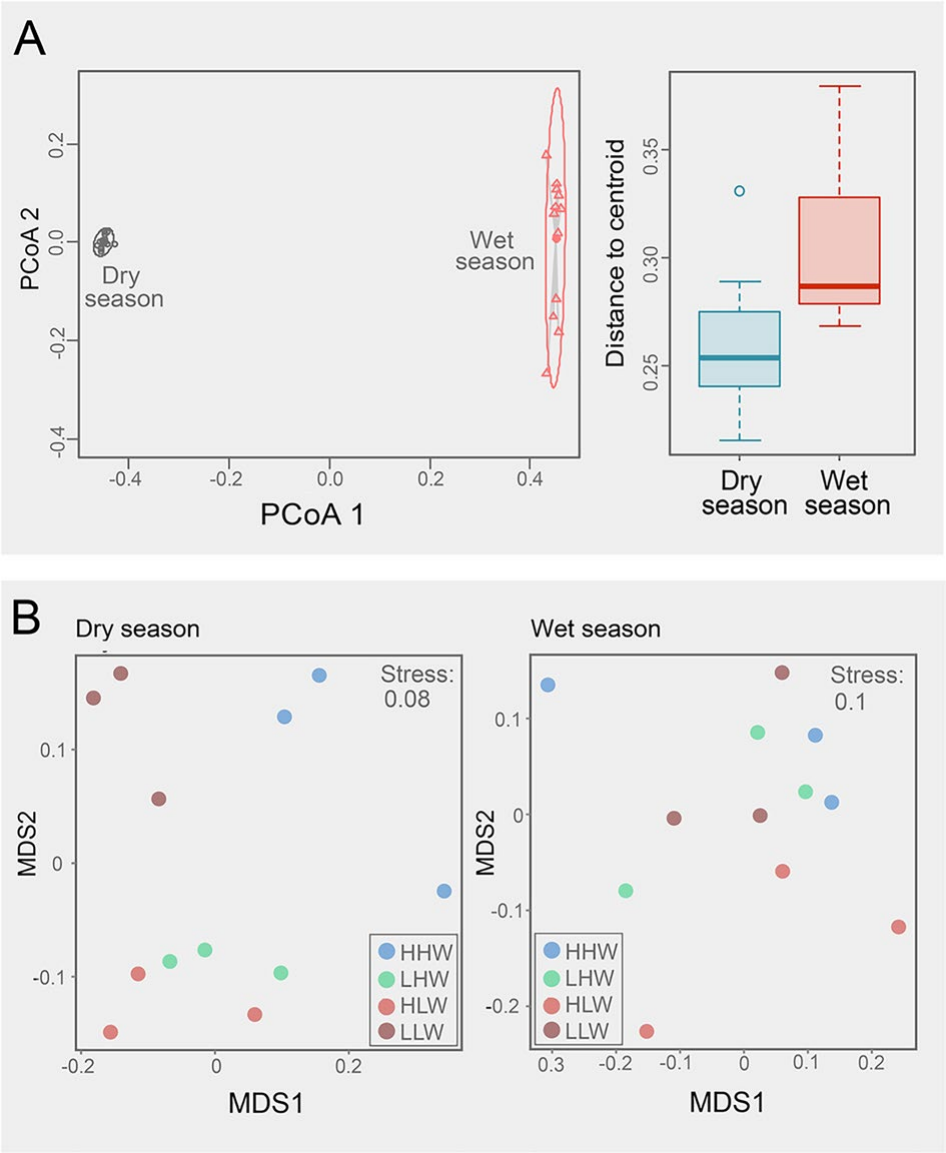
Multivariate homogeneity of group dispersion according to season (**A**). Nonmetric multidimensional scaling (NMDS) analysis based on Bray–Curtis dissimilarity among tidal levels (**B**). *HHW* higher high water, *LHW* lower low water, *HLW* higher low water, *LLW* lower low water

Multivariate homogeneity analysis indicated a lower dispersion around the median in the dry season than that in the wet one (Fig. 3A; average distance to centroids, dry = 0.2597, wet = 0.3031), and a significant difference was detected between seasons (permutation test, *p* < 0.01). Further, the community composition significantly differed along the diurnal variation of the tide only during the dry season (ANOSIM for dry season, *R*: 0.68, *p* < 0.001; ANOSIM for the wet season, *R*: 0.08, *p* = 0.24). Based on Bray–Curtis dissimilarity, the ordination analysis showed that during the dry season, bacterial communities from HHW and LLW were segregated compared with the other tidal levels (Fig. 3B). Instead, no segregation was observed during the wet season. Water temperature (*R*^2^ = 0.59), salinity (*R*^2^ = 0.88), and total dissolved solids (*R*^2^ = 0.88) were significant factors (Supplementary Table S2; *p* < 0.05) explaining community segregation along the tidal fluctuations during the dry season, but not in the wet one.

**Fig. 4:**
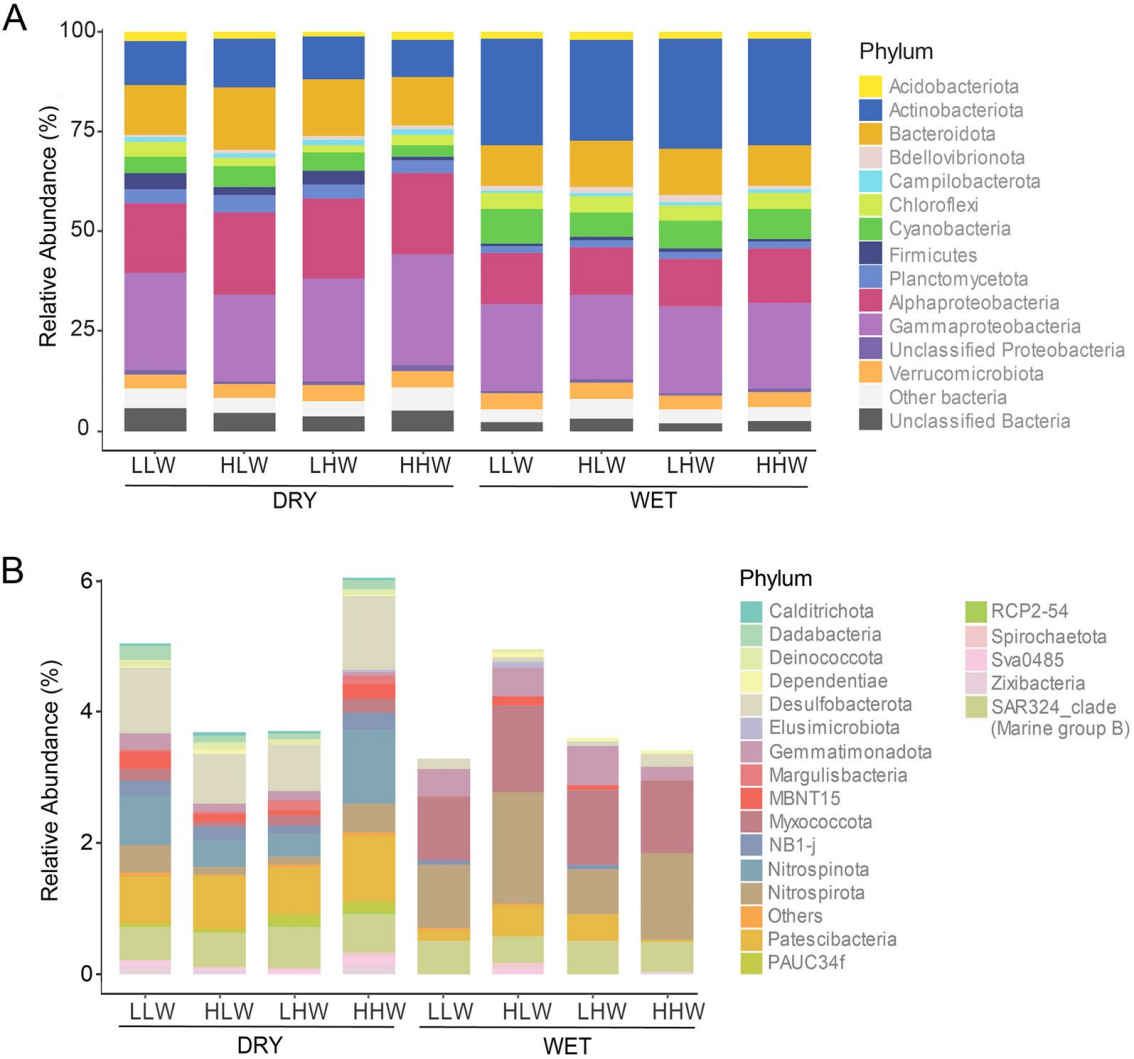
Mean relative abundance of phyla > 1% (**A**) and < 1% (**B**) for the different tidal levels. *HHW* higher high water, *LHW* lower low water, *HLW* higher low water, *LLW* lower low water

The most abundant phyla were Proteobacteria (33.8–49.6%; primarily Alphaproteobacteria and Gammaproteobacteria), Actinobacteriota (9.25–27.6%), Bacteroidota (10–15.7%), and Cyanobacteria (2.8–8.8%). Although the main bacterial abundant groups were the same among the four tidal levels (Supplementary Data File 1), their relative contribution changed between seasons (Fig. 4A). For example, the mean relative abundance of Actinobacteria (dry = 9.2–12.3%; wet = 25.3–27.6%) and Cyanobacteria (dry = 2.8–5.2%; wet = 5.9–8.8%) was higher in the wet season. Instead, Bacteroidetes (dry = 12.2–15.7%; wet = 10–11.5%), Firmicutes (dry = 1–4%; wet = 0.4–0.84%), and Alphaproteobacteria (dry = 17.2–20.8%; wet = 11.8–13.8%) were slightly higher in the dry one. Marked differences in bacterial groups were found when phyla with a mean relative abundance < 1% were compared (Fig. 4B). In this analysis, Calditrichota (0.04–0.05%), Dadabacteria (0.08–0.2%), Margulisbacteria (0.04–0.17%), Nitrospinota (0.36–1.1%), PAUC34f (0.048–1.6%), and RCP2-54 (0.03–0.04%) were found only in the dry season. Fusobacteriota (only in LLW) and TX1A-33 (only in HLW) were the phyla only detected during the wet season. Some bacterial groups denominated as “others” in Fig. 4B were detected in specific tidal levels. For example, DTB120 (0.02%) and WS2 (0.03%) were detected only in LLW, WPS-2 (0.03%) in LHW, and Synergistota (0.02%) and Deferrisomatota 0.04%) in HHW in the dry season. In the wet one, Fusobacteriota (0.04%) and TX1A-33 (0.04%) were detected in LLW and HLW, respectively.

The relative abundance of some bacterial groups showed significant differences among tidal levels (Supplementary Fig. 2). For example, the high tidal levels in the dry season showed a significantly high relative abundance of Campylobacterales during LHW and Rhodospirillales and unclassified groups during the HHW. In the low tidal levels, Rhizobiales, Rhodobacterales, and unclassified sequences within the UBA10353 marine group were significant for HLW, whereas Clostridia (Firmicutes) were significant for LLW. During the wet season, certain bacterial groups demonstrated a significantly higher relative abundance, but this was only observed during periods of high tidal levels. Specifically, the bacterial groups Oligoflexia and Frankiales showed increased abundance at low high water (LHW) tides, while Acidimicrobiia showed increased abundance at high water (HHW) tides.

**Fig. 5:**
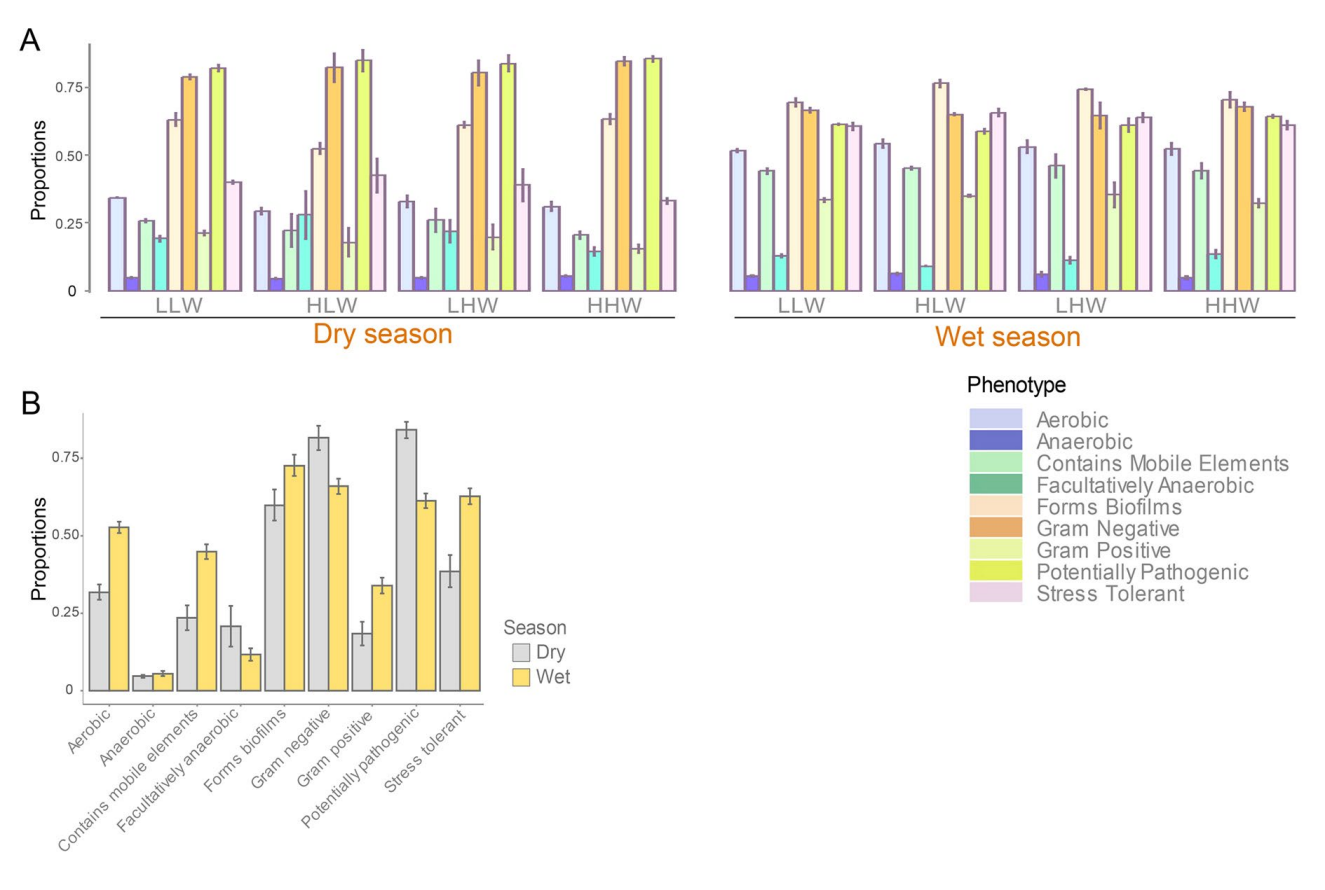
Proportions of predicted phenotypes among tidal levels (**A**) and between seasons (**B**). *HHW* higher high water, *LHW* lower low water, *HLW* higher low water, *LLW* lower low water

The main predicted phenotypes were related to potential pathogenicity, gram-negative, and biofilm formation traits (Fig. 5). Tidal levels showed similar proportions of predicted phenotypes within each season and thus, significant differences (Mann–Whitney–Wilcoxon test, *p* > 0.05) were not detected among them (Fig. 5A). Gram-negative (81% ± 4%), facultatively anaerobic (20% ± 7%), and potentially pathogenic prokaryotes (84% ± 2%) had higher proportions during the dry season (Fig. 5B). Instead, microorganisms classified as gram-positive (34% ± 3%), forming biofilm (73% ± 3%), containing mobile elements (45% ± 2%), aerobic (53% ± 2%), and tolerant to stress (63% ± 3%) had higher proportions in the wet season. In fact, a significant difference (Kruskal–Wallis test, *p* < 0.05) was detected when phenotypes were compared between seasons (dry vs. wet). Few phyla contributed to the main phenotypes. For example, potentially pathogenic microorganisms were related to Proteobacteria, Cyanobacteria, and Actinobacteria (only in the dry season). Bacteria potentially forming biofilms were primarily related to Proteobacteria and Actinobacteria, but differences between season and tidal levels were not observed.

**Fig. 6:**
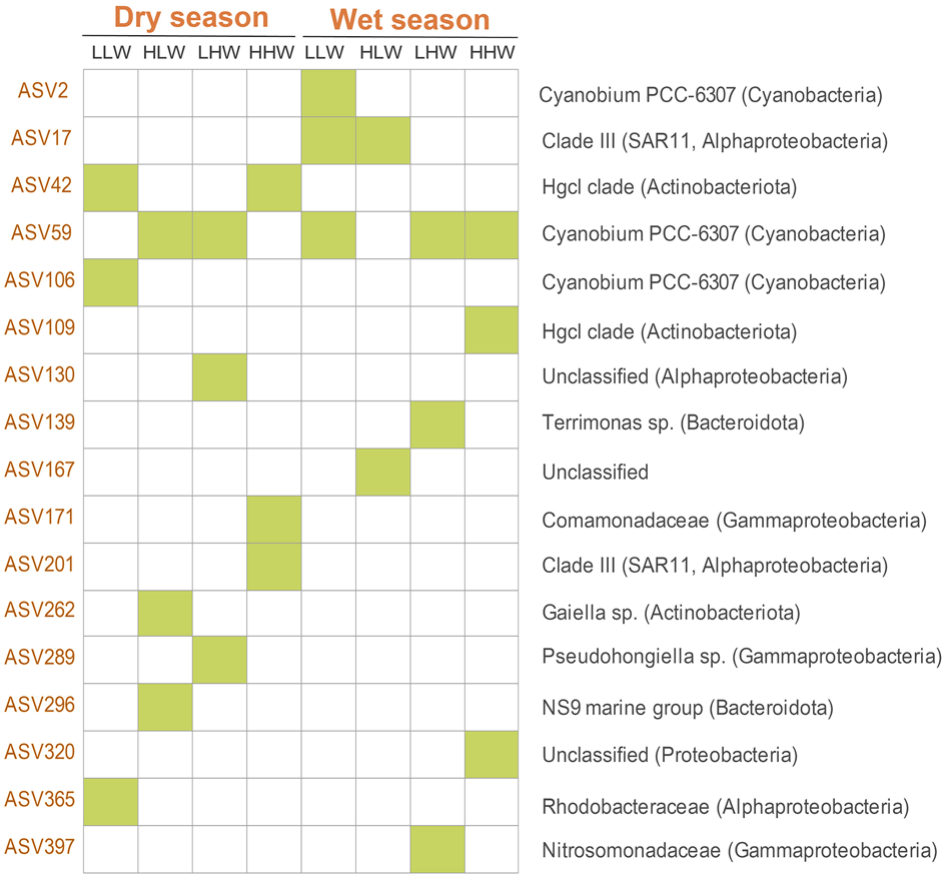
Bacterial indicators of each tidal level during both seasons. The candidate taxa are those that fulfill the requirement of being present in a minimum of 80% of the samples at each tidal level

To assess the specific association of taxa with different tidal levels, we evaluated bacterial indicators. These indicators were based on the presence of three ASVs for most tidal levels, except for HLW during the wet season, which had only two ASVs. Indicators were found in Cyanobacteria, Actinobacteriota, Bacteroidota, and Proteobacteria (Fig. 6). In addition, one indicator remained unclassified at the phylum level. In the dry season, HLW had ASV59, ASV262, and ASV296, while LHW contained ASV59, ASV130, and ASV289. LLW showed ASV42, ASV106, and ASV365, and HHW displayed ASV42, ASV171, and ASV201. In the wet season, LLW exhibited ASV17, ASV2, and ASV59, while HHW presented ASV59, ASV109, and ASV320. HLW was characterized by ASV17 and ASV167, while LHW showed ASV59, ASV139, and ASV397. ASV59 exhibited the highest presence across various tidal levels, corresponding to the cyanobacterium *Cyanobium* PCC-6307. Furthermore, multiple indicators were associated with this cyanobacterium (ASV2, ASV59, and ASV106). Other indicators were represented by Proteobacteria (gamma and alpha), Actinobacteriota, Bacteroidota, and unclassified.

## Discussion

Estuaries provide numerous ecosystem services, such as coastal protection, erosion control, maintenance of fisheries, and carbon sequestration (Barbier et al. 2011). They are extremely dynamic ecosystems where fresh and marine waters interact to cause a high spatial and temporal variability in physicochemical parameters to which organisms have to adapt (McLusky 1993). For example, gradients mainly of salinity affect growth rates and taxonomical composition of microbial components along estuaries (Bouvier and del Giorgio 2002; Campbell and Kirchman 2013; Crump et al. 2004). Here, we found that the bacterial community composition and diversity significantly change depending on the diurnal tidal dynamics during the dry season. By contrast, these changes were less pronounced during the wet season. Our study encompassed both the genotypic aspects, such as community composition and the phenotypic trait prediction, thus showing how these bacteria potentially adapt to tidal and seasonal variations.

The typical high variability of microbial diversity between seasons in estuaries (Marín‑Vindas et al. 2023) was also detected in our study. For example, diversity metrics did not show specific trends among tidal levels and between seasons (Fig. 2A). Samples grouped within the dry season had significantly higher richness and phylogenetic diversity values, but samples from the different tidal levels within the wet season had significantly higher inverse Simpson values (Fig. 2B). This can be interpreted as bacteria having high abundance but low diversity during the dry season, with the opposite trend in the wet one. When tidal levels were analyzed separately, the lower low water level (LLW) of the dry season had the highest values in all diversity metrics (Fig. 2). This maximum could be related to the resuspension of sediments (TDS: 19.2 g/L) and their associated bacterial community. We anticipated minimal variations in the bacterioplankton community composition across different tidal levels during the wet season. This expectation is rooted in the substantial higher water discharge during this period, which likely leads to a homogenization of microbial communities. Our findings, as corroborated by the ANOSIM test (*R*: 0.08, *p* = 0.24), align with this hypothesis. Conversely, the dry season presented a contrasting scenario. With reduced freshwater flow and increased saltwater intrusion, there was a pronounced differentiation in the microbial communities among tidal levels, a distinction that was statistically significant as evidenced by the ANOSIM results (*R*: 0.68, *p* < 0.001).

The bacterial community composition of the upper estuary of the Bangpakong River resembled typical estuarine communities found in similar ecosystems, with Proteobacteria as the most important phylum (Ghosh and Bhadury 2019; Marín‑Vindas et al. 2023). For example, within this phylum, Gammaproteobacteria dominated in the estuaries Mooriganga (India), Thakuran (India), Matla (India), Harinbhanga (India), Jiulong (China), and Columbia (United States) (Ghosh and Bhadury 2019), whereas Alphaproteobacteria dominated in the Delaware (United States), Hangzhou (China), and Pearl (China) ones (Campbell and Kirchman 2013; Ghosh and Bhadury 2019). However, the prevalence of these proteobacterial classes could be linked to specific seasons, as exemplified by the estuary of the Gulf of Nicoya (Costa Rica), where Gammaproteobacteria dominate during the dry season, while Alphaproteobacteria do so during the wet one (Marín‑Vindas et al. 2023). In our study, Gammaproteobacteria was the most abundant class, accounting for 21.2–27.9% of the entire community, followed by Alphaproteobacteria that represented between 11.8 and 20.8% of the community. Among the Gammaproteobacteria, unclassified members held the highest relative abundance during the dry season, while Burkholderiales emerged as the dominant group during the wet season. The dominance of Burkholderiales was probably related to the large export of organic matter of fluvial and terrestrial origin to the gulf of Thailand during the wet season (Bordalo et al. 2016). This group is known to be found in freshwaters, but representatives usually derive from the terrestrial catchment (Ruiz‑González et al. 2015). By contrast, members of Oceanospirillales, another important order among Proteobacteria, were present in high relative abundance only in the dry season when seawater intrusions are dominant. Although members of this order are important DIC fixers (DeLorenzo et al. 2012) and capable of thriving in a wide range of organic carbon sources (Mou et al. 2008), their low abundance in the wet season could be related to their apparent inability to thrive in the presence of terrestrial DOM (Sipler et al. 2017).

In general, the bacterial community composition in the upper estuary of Bangpakong River was largely influenced by the seawater intrusion during the dry season and by the freshwater discharge during the wet one. This was reflected by one of the dominant bacterial groups, the Alphaproteobacteria. This class was mainly composed of Rhodobacterales, SAR11 clade, and Rhizobiales. The first two orders were more abundant during the dry season and are considered typical marine bacteria in estuaries (Kieft et al. 2018). By contrast, Rhizobiales, with a higher abundance in the wet season, are considered core root microbiota of different soil types (Garrido‑Oter et al. 2018; Yeoh et al. 2017). The effect of the seawater intrusion was also observed when considering the rare phyla. For example, phyla such as Calditrichota, Dadabacteria, and Margulisbacteria were detected only in the dry season. These phyla are abundant in seawater and marine sediments (Begmatov et al. 2021; Graham and Tully 2021; Marshall et al. 2017; Matheus Carnevali et al. 2019), and their presence suggests a higher mixture of water and sediment in this season.

The Bangpakong River basin encompasses diverse land uses, extending from seasonal rice fields and various annual and perennial agricultural endeavors to rubber plantations and forested areas, including tropical hardwoods and mangrove swamps. It also supports the livelihood of a wide range of communities involved in agro-forestry, agriculture, and fisheries (Sangmanee et al. 2013). All these land uses and human activities have largely affected the river’s water quality (Bundao et al. 2018). In fact, previous studies showed that the water quality (based on chemical and biological parameters) of different sections of this river was low, especially during the dry season (Bordalo et al. 2001). Our results also provide evidence that water quality is probably lower during the dry season than in the wet one. For example, the presence of predicted potential pathogens during the dry season reached up to 84% of the whole community, and typical pathogens such as the ones within Vibrionales (0.23–1%), Clostridia (0.48–1.6%), and Mycobacteriaceae (0.48–1%) were found. Contamination with organic nitrogen in estuaries could promote blooms of potentially pathogenic Vibrionales (Gutiérrez‑Barral et al. 2021), such as *Vibrio cholerae,* that produce a severe diarrheal disease (Camberg and Sandkvist 2005). Further, Clostridia produce many toxins responsible for severe infections in humans and animals (Popoff and Bouvet 2013). Mycobacteriaceae contain some of the most significant human pathogens (e.g., *Mycobacterium tuberculosis* and *M. leprae*) (Gupta 2021). Further, the LEfSe algorithm (Supplementary Fig. S2) also identified some pathogenic groups in the dry season, namely Clostridia and Bacilli. The latter group consists of spore-forming bacteria, with some members being well-known pathogens (Liu et al. 2021). Campylobacterota was also identified as a significant group at LHW in the dry season. This phylum is composed of spiral-shaped motile bacteria which include representatives recognized as important human pathogens, such as *Helicobacter pylori* and *Campylobacter jejuni* (van der Stel and Wösten 2019).

Despite the lack of a clear differentiation of bacterial indicators among different tidal levels, these indicators provide valuable insights into bacterial associations within specific tidal levels during both dry and wet seasons. Notably, one such indicator is ASV59, a taxon of Cyanobacteria, which consistently appears as a strong indicator for most of the tidal levels and suggests its ability to thrive in diverse environmental conditions. The consistent presence of ASV59 (Cyanobium PCC-6307) throughout both wet and dry seasons highlights its resilience and potential significance within the studied ecosystem. Given the well-established sensitivity of estuarine microbial communities to changes in the watershed (Alonso et al. 2022; Marín‑Vindas et al. 2023), it is plausible that shifts in bacterial composition and prevalence could reflect diffuse and point pollution inputs. The freshwater picocyanobacterium *Cyanobium*, particularly *Cyanobium* PCC-6307 (Shih et al. 2013), is not typically associated with toxicity or pathogenicity. However, this taxon has the potential to produce harmful compounds that inhibit the growth of co-occurring bacteria, which could confer it a competitive advantage in certain environments (Costa et al. 2015). Furthermore, picoplanktonic cyanobacteria, including certain strains of *Cyanobium*, have been implicated in microcystin production, highlighting the potential health risks they might pose to both animals and humans (Silva et al. 2014). Intriguingly, in a separate investigation, *Cyanobium* PCC-6307 was found to coincide with elevated levels of *Vibrio parahaemolyticus* in water, suggesting a potential association or shared environmental preference between this cyanobacterium and the pathogenic *Vibrio* (Diner et al. 2023). While the presence of *Cyanobium* PCC-6307 in a contaminated river might initially seem benign given its general non-toxic nature, these findings underscore the importance of considering the broader ecological and health implications of such occurrences, especially considering emerging evidence. Exploring additional indicators representing Proteobacteria (Gamma and Alpha), Bacteroidota, and Actinobacteriota could provide insights into their potential roles in responding to varying levels of pollutants.

## Conclusions

Our study in the upper estuary of the Bangpakong River, conducted across various tidal levels during both dry and wet seasons, demonstrates how daily tidal patterns affect bacterial communities. Understanding the microbial composition at different tidal levels is crucial for appreciating local biodiversity and for accurately assessing the water quality of this heavily impacted aquatic ecosystem. Furthermore, it is essential to extend the study from the river mouth to the lower estuary to explore how microbial communities adapt to varying levels of urbanization and the type of land use. Integrating the findings of this study with pollution-monitoring data in future research could enhance our understanding of the impact of different industries and human activities on lotic ecosystems and how microbial communities differ between tidal and non-tidal river systems.

## Supplementary Information

Below is the link to the electronic supplementary material.

Supplementary file1 (DOCX 929 KB)

## Data Availability

Raw amplicon reads were deposited into NCBI’s Sequence Read Archive (SRA) under accession number GSE85337 and are available at the following URL: https://www.ncbi.nlm.nih.gov/bioproject/?term=PRJNA977079.

## References

[CR1] Alonso C, Pereira E, Bertoglio F, De Cáceres M, Amann R (2022) Bacterioplankton composition as an indicator of environmental status: proof of principle using indicator value analysis of estuarine communities. Aquat Microb Ecol 88:1–18

[CR2] Astudillo-García C, Hermans SM, Stevenson B, Buckley H, Lear G (2019) Microbial assemblages and bioindicators as proxies for ecosystem health status: potential and limitations. Appl Microbiol Biotechnol 103:6407–6421 31243501 10.1007/s00253-019-09963-0

[CR3] Barbier EB, Hacker SD, Kennedy C, Koch EW, Stier C, Silliman R (2011) The value of estuarine and coastal ecosystem services. Ecol Monogr 81:169–193

[CR4] Battin TJ, Besemer K, Bengtsson MM, Romani AM, Packmann A (2016) The ecology and biogeochemistry of stream biofilms. Nat Rev Microbiol 14:251–263 26972916 10.1038/nrmicro.2016.15

[CR5] Begmatov S, Savvichev AS, Kadnikov VV, Beletsky AV, Rusanov II, Klyuvitkin AA, Novichkova EA, Mardanov AV, Pimenov NV, Ravin NV (2021) Microbial communities involved in methane, sulfur, and nitrogen cycling in the sediments of the Barents sea. Microorganisms 9:2362 34835487 10.3390/microorganisms9112362 PMC8625253

[CR6] Boonphakdee T, Sawangwong P, Fujiwara T (1999) Freshwater discharge of Bangpakong River flowing into the inner Gulf of Thailand. La Mer 37:103–109

[CR7] Boonphakdee T, Kasai A, Fujiwara T, Sawangwong P, Cheevaporn V (2008) Combined stable carbon isotope and c/n ratios as indicators of source and fate of organic matter in the Bangpakong River Estuary, Thailand. EnvironmentAsia 1:28–36

[CR8] Bordalo AA, Nilsumranchit W, Chalermwat K (2001) Water quality and uses of the Bangpakong River (eastern Thailand). Water Res 35:3635–3642 11561624 10.1016/s0043-1354(01)00079-3

[CR9] Bordalo AA, Chalermwat K, Teixeira C (2016) Nutrient variability and its influence on nitrogen processes in a highly turbid tropical estuary (Bangpakong, Gulf of Thailand). J Environ Sci 45:131–142 10.1016/j.jes.2016.01.011 27372127

[CR10] Bouvier TC, del Giorgio PA (2002) Compositional changes in free-living bacterial communities along a salinity gradient in two temperate estuaries. Limnol Oceanogr 47:453–470

[CR11] Bundao S, Veeravaitaya N, Kaewnern M, Ingthamjitr S (2018) The relationship between land use and water quality in Bangpakong Estuary, Thailand. J Fish Environ 42:24–31

[CR12] Cáceres M, Legendre P (2009) Associations between species and groups of sites: indices and statistical inference. Ecology 90:3566–3574 20120823 10.1890/08-1823.1

[CR13] Callahan BJ, McMurdie PJ, Rosen MJ, Han AW, Johnson A, Holmes S (2016) DADA2: High-resolution sample inference from Illumina amplicon data. Nat Methods 13:581–583 27214047 10.1038/nmeth.3869 PMC4927377

[CR14] Camberg JL, Sandkvist M (2005) Molecular analysis of the *Vibrio cholerae* type II secretion ATPase EpsE. J Bacteriol 187:249–256 15601709 10.1128/JB.187.1.249-256.2005 PMC538811

[CR15] Campbell BJ, Kirchman DL (2013) Bacterial diversity, community structure and potential growth rates along an estuarine salinity gradient. ISME J 7:210–220 22895159 10.1038/ismej.2012.93 PMC3526181

[CR16] Caporaso JG, Lauber CL, Walters WA, Berg-Lyons D, Lozupone CA, Turnbaugh PJ, Fierer N, Knight R (2011) Global patterns of 16S rRNA diversity at a depth of millions of sequences per sample. Proc Nat Acad Sci 108:4516–4522 20534432 10.1073/pnas.1000080107 PMC3063599

[CR17] Chen W, Ren K, Isabwe A, Chen H, Liu M, Yang J (2019a) Correction to: Stochastic processes shape microeukaryotic community assembly in a subtropical river across wet and dry seasons. Microbiome 7:148 31727140 10.1186/s40168-019-0763-x PMC6857158

[CR18] Chen X, Wei W, Wang J, Li H, Sun J, Ma R, Jiao N, Zhang R (2019b) Tide driven microbial dynamics through virus-host interactions in the estuarine ecosystem. Water Res 160:118–129 31136846 10.1016/j.watres.2019.05.051

[CR19] Clark DR, McKew BA, Binley A, Heppell C, Whitby C, Trimmer M (2022) Hydrological properties predict the composition of microbial communities cycling methane and nitrogen in rivers. ISME Commun 2:5 37938696 10.1038/s43705-022-00087-7 PMC9723640

[CR20] Costa MS, Costa M, Ramos V, Leao PN, Barreiro A, Vasconcelos V, Martins R (2015) Picocyanobacteria from a clade of marine Cyanobium revealed bioactive potential against microalgae, bacteria, and marine invertebrates. J Toxicol Environ Health Part A 78:432–442 10.1080/15287394.2014.991466 25785557

[CR21] Crump BC, Hopkinson CS, Sogin ML, Hobbie JE (2004) Microbial biogeography along an estuarine salinity gradient: combined influences of bacterial growth and residence time. Appl Environ Microbiol 70:1494–1505 15006771 10.1128/AEM.70.3.1494-1505.2004 PMC365029

[CR22] Day JW, Crump BC, Kemp WM, Yáñez-Arancibia A (eds) (2012) Estuarine ecology. Wiley, Hoboken

[CR23] De Boer PL, Oost AP, Vi MJ (1989) The diurnal inequality of the tide as a parameter for recognizing tidal influences. Sepm Jsr 59:912–921

[CR24] DeLorenzo S, Bräuer SL, Edgmont CA, Herfort L, Tebo BM, Zuber P (2012) Ubiquitous dissolved inorganic carbon assimilation by marine bacteria in the Pacific Northwest coastal ocean as determined by stable isotope probing. PLoS ONE 7:e46695 23056406 10.1371/journal.pone.0046695 PMC3463544

[CR25] Diner RE, Zimmer-Faust A, Cooksey E, Allard S, Kodera SM, Kunselman E, Garodia Y, Verhougstraete MP, Allen AE, Griffith J, Gilbert J (2023) Host and water microbiota are differentially linked to potential human pathogen accumulation in oysters. Appl Environ Microbiol 89:e0031823 37318344 10.1128/aem.00318-23 PMC10370324

[CR26] Elliott M, McLusky DS (2002) The need for definitions in understanding estuaries. Estuar Coast Shelf Sci 55:815–827

[CR27] Ensign SH, Doyle MW, Piehler MF (2013) The effect of tide on the hydrology and morphology of a freshwater river. Earth Surf Process Landforms 38:655–660

[CR28] Faith DP (1992) Conservation evaluation and phylogenetic diversity. Biol Conserv 61:1–10

[CR29] Findlay S (2010) Stream microbial ecology. J N Am Benthol Soc 29:170–181

[CR02] Fine PV, Kembel SW (2011) Phylogenetic community structure and phylogenetic turnover across space and edaphic gradients in western Amazonian tree communities. Ecography 34:552–565

[CR30] Garrido-Oter R, Nakano RT, Dombrowski N, Ma KW, Agbiome MAC, Schulze-Lefert P (2018) Modular traits of the rhizobiales root microbiota and their evolutionary relationship with Symbiotic Rhizobia. Cell Host Microbe 24:155–167 30001518 10.1016/j.chom.2018.06.006 PMC6053594

[CR31] Ghosh A, Bhadury P (2019) Exploring biogeographic patterns of bacterioplankton communities across global estuaries. Microbiologyopen 8:e00741 30303297 10.1002/mbo3.741 PMC6528645

[CR32] Gong F, Huang S, Xie W, Zhang H, Lan F, Yin K (2024) Effects of tidal cycles on the variability of microbial communities in a semiclosed bay. Cont Shelf Res 272:105147

[CR33] Graham ED, Tully BJ (2021) Marine Dadabacteria exhibit genome streamlining and phototrophy-driven niche partitioning. ISME J 15:1248–1256 33230264 10.1038/s41396-020-00834-5 PMC8115339

[CR34] Guizien K, Dupuy C, Ory P, Montanie H, Hartmann H, Chatelain M, Karpyychev M (2014) Microorganism dynamics during a rising tide: disentangling effects of resuspension and mixing with offshore waters above an intertidal mudflat. J Mar Syst 129:178–188

[CR35] Gupta RS (2021) Microbial taxonomy: how and why name changes occur and their significance for (clinical) microbiology. Clin Chem 68:134–137 34969111 10.1093/clinchem/hvab188

[CR36] Gutiérrez-Barral A, Teira E, Hernández-Ruiz M, Fernandez E (2021) Response of prokaryote community composition to riverine and atmospheric nutrients in a coastal embayment: role of organic matter on Vibrionales. Estuar Coast Shelf Sci 251:107196

[CR37] Herlemann D, Labrenz M, Jürgens K, Bertilsson S, Waniek J, Andersson A (2011) Transitions in bacterial communities along the 2000 km salinity gradient of the Baltic Sea. ISME J 5:1571–1579 21472016 10.1038/ismej.2011.41 PMC3176514

[CR38] Hoitink AJF, Jay DA (2016) Tidal river dynamics: implications for deltas. Rev Geophys 54:240–272

[CR39] Jovanovic D, Coleman R, Deletic A, Maccarthy D (2017) Tidal fluctuations influence *E. coli* concentrations in urban estuaries. Mar Pollut Bull 119:226–230 28396075 10.1016/j.marpolbul.2017.04.004

[CR40] Kieft B, Li Z, Bryson S, Crump B, Hettich R, Pan C, Mayali X, Mueller R (2018) Microbial community structure-function relationships in Yaquina Bay estuary reveal spatially distinct carbon and nitrogen cycling capacities. Front Microbiol 9:1282 29963029 10.3389/fmicb.2018.01282 PMC6010575

[CR41] Langille MGI, Zaneveld J, Caporaso JG, McDonald D, Knights D, Reyes JA, Clemente JC, Burkepile DE, Vega TRL, Knight R, Beiko RG, Huttenhower C (2013) Predictive functional profiling of microbial communities using 16S rRNA marker gene sequences. Nat Biotechnol 31:814–821 23975157 10.1038/nbt.2676 PMC3819121

[CR42] Lee M, Park BS, Baek SH (2018) Tidal influences on biotic and abiotic factors in the Seomjin River Estuary and Gwangyang Bay, Korea. Estuar Coast 41:1–17

[CR43] Liu J, Luo Y, Xu Z, Kjellerup B (2021) Food pathogens. In: Galanakis CM (ed) Innovative food analysis. Elsevier, pp 295–321

[CR44] Love MI, Huber W, Anders S (2014) Moderated estimation of fold change and dispersion for RNA-seq data with DESeq2. Genome Biol 15:550 25516281 10.1186/s13059-014-0550-8 PMC4302049

[CR45] Ma Y, Chanpiwat P (2022) A case study on the correlation between land use types and water quality of the Bang Pakong River in Chachoengsao Province. Highl Sci Eng Technol 20:102–108

[CR46] Marín-Vindas C, Sebastián M, Ruiz-González C, Balague V, Vega-Corrales L, Gasol JM (2023) Shifts in bacterioplankton community structure between dry and wet seasons in a tropical estuary strongly affected by riverine discharge. Sci Total Environ 903:166104 37558065 10.1016/j.scitotenv.2023.166104

[CR47] Markowitz VM, Chen I-MA, Palaniappan K, Palaniappan K, Chu K, Szeto E, Pillay M, Ratner A, Huang J, Woyke T, Huntemann M, Anderson I, Billis K, Varghese N, Mavromatis K, Pati A, Ivanova NN, Kyrpides N (2014) IMG 4 version of the integrated microbial genomes comparative analysis system. Nucleic Acids Res 42:D560–D567 24165883 10.1093/nar/gkt963 PMC3965111

[CR48] Marshall IPG, Starnawski P, Cupit C, Fernandez-Caceres E, Ettema T, Schramm A, Kjeldsen KU (2017) The novel bacterial phylum Calditrichaeota is diverse, widespread and abundant in marine sediments and has the capacity to degrade detrital proteins. Environ Microbiol Rep 9:397–403 28488795 10.1111/1758-2229.12544

[CR49] Matheus-Carnevali PB, Schulz F, Castelle CJ, Kantor RS, Shih PM, Sharon I, Santini JM, Olm MR, Amano Y, Thomas BC, Anantharaman K, Burstein D, Becraft ED, Stepanauskas R, Woyke T, Banfield J (2019) Hydrogen-based metabolism as an ancestral trait in lineages sibling to the Cyanobacteria. Nat Commun 10:463 30692531 10.1038/s41467-018-08246-y PMC6349859

[CR50] McLellan SL, Eren AM (2014) Discovering new indicators of fecal pollution. Trends Microbiol 22:697–706 25199597 10.1016/j.tim.2014.08.002 PMC4256112

[CR51] McLusky DS (1993) Marine and estuarine gradients—an overview. Neth J Aquat Ecol 27:489–493

[CR52] Moftakhari HR, Jay DA, Talke SA, Kukulka T, Bromirski PD (2013) A novel approach to flow estimation in tidal rivers. Water Resour Res 49:4817–4832

[CR53] Mou X, Sun S, Edwards RA, Hodson R, Moran MA (2008) Bacterial carbon processing by generalist species in the coastal ocean. Nature 451:708–711 18223640 10.1038/nature06513

[CR54] Murali A, Bhargava A, Wright ES (2018) IDTAXA: a novel approach for accurate taxonomic classification of microbiome sequences. Microbiome 6:140 30092815 10.1186/s40168-018-0521-5 PMC6085705

[CR55] Murray KS, Fisher LE, Therrien J, George B, Gillespie J (2001) Assessment and use of indicator bacteria to determine sources of pollution to an urban river. J Great Lakes Res 27:220–229

[CR56] O’Connor JA, Erler DV, Ferguson A, Maher DT (2022) The tidal freshwater river zone: Physical properties and biogeochemical contribution to estuarine hypoxia and acidification—the “hydrologic switch.” Estuar Coast Shelf Sci 268:107786

[CR57] Oksanen J, Simpson GL, Blanchet FG, Kindt R, Legendre P, Minchin PR, Ohara RB, Solymos P, Stevens MHH, Szoecs E, Wagner H, Barbour M, Bedward M, Bolker B, Borcard D, Carvalho G, Chirico M, De Caceres M, Durand S, Antoniazi HB et al (2019) Vegan: Community Ecology Package., R Package Version 2.0-7. https://cran.r-project.org/web/packages/vegan/index.html. Accessed 29 Apr 2019

[CR58] Okwala T, Shrestha S, Ghimire S, Mohanasundaram S, Datta A (2020) Assessment of climate change impacts on water balance and hydrological extremes in Bang Pakong-Prachin Buri river basin, Thailand. Environ Res 186:109544 32361258 10.1016/j.envres.2020.109544

[CR60] Popoff MR, Bouvet P (2013) Genetic characteristics of toxigenic Clostridia and toxin gene evolution. Toxicon 75:63–89 23707611 10.1016/j.toxicon.2013.05.003

[CR61] Pusch M, Fiebig D, Brettar I, Eisenmann H, Ellis BK, Kaplan LA, Lock MA, Naegeli MW, Traunspurger W (1998) The role of micro-organisms in the ecological connectivity of running waters. Freshw Biol 40:453–495

[CR62] Raymond PA, Hartmann J, Lauerwald R, Sobek S, McDonald C, Hoover M, Butman D, Striegl R, Mayorga E, Humborg C, Kortelainen P, Durr H, Meybeck M, Ciais P, Guth P (2013) Global carbon dioxide emissions from inland waters. Nature 503:355–359 24256802 10.1038/nature12760

[CR63] Ruiz-González C, Niño-García JP, Del Giorgio PA (2015) Terrestrial origin of bacterial communities in complex boreal freshwater networks. Ecol Lett 18:1198–1206 26306742 10.1111/ele.12499

[CR64] Sangmanee C, Wattayakorn G, Sojisuporn P (2013) Simulating changes in discharge and suspended sediment loads of the. Maejo Int J Sci Technol 7:72–84

[CR65] Sassi MG, Hoitink AJF (2013) River flow controls on tides and tide-mean water level profiles in a tidal freshwater river. J Geophys Res Oceans 118:4139–4151

[CR01] Savio D, Sinclair L, Ijaz UZ, Parajka J, Reischer GH, Stadler P, Blaschke AP, Blöschl G, Mach RL, Kirschner AK, Farnleitner AH (2015) Bacterial diversity along a 2600 km river continuum. Environ Microbiol 17:4994–5007 25922985 10.1111/1462-2920.12886 PMC4918796

[CR66] Segata N, Izard J, Waldron L, Gevers D, Miropolsky L, Garret W, Huttenhower C (2011) Metagenomic biomarker discovery and explanation. Genome Biol 12:R60 21702898 10.1186/gb-2011-12-6-r60 PMC3218848

[CR67] Shih PM, Wu D, Latifi A, Axen SD, Fewer DP, Talla E, Calteau A, Cai F, Tandeau de Marsac N, Rippka R, Herdman M, Sivonen CT, Laurent T, Goodwin L, Nolan M, Davenport KW, Han CS, Rubin EM, Eisen JA et al (2013) Improving the coverage of the cyanobacterial phylum using diversity-driven genome sequencing. Proc Natl Acad Sci USA 110:1053–1058 23277585 10.1073/pnas.1217107110 PMC3549136

[CR68] Silva CSP, Genuário DB, Vaz MG, Fiore MF (2014) Phylogeny of culturable cyanobacteria from Brazilian mangroves. Syst Appl Microbiol 37:100–112 24461713 10.1016/j.syapm.2013.12.003

[CR69] Sipler RE, Kellogg CTE, Connelly TL, Roberts QN, Yager PL, Bronk DA (2017) Microbial community response to terrestrially derived dissolved organic matter in the coastal arctic. Front Microbiol 8:1018 28649233 10.3389/fmicb.2017.01018 PMC5465303

[CR70] Snyder EE, Kampanya N, Lu J, Nordberg EK, Karur HR, Shukla M, Soneja J, Xue T, Yoo H, Zhang F, Dharmanolla C, Dongre NV, Gillespie JJ, Hamelius J, Hance M, Huntington KI, Jukneliene D, Koziski J, Mackasmiel L, Mane SP (2007) PATRIC: the VBI pathosystems resource integration center. Nucleic Acids Res 35:D401–D406 17142235 10.1093/nar/gkl858 PMC1669763

[CR71] Spietz RL, Williams CM, Rocap G, Horner-Devine MC (2015) A dissolved oxygen threshold for shifts in bacterial community structure in a seasonally hypoxic estuary. PLoS ONE 10:e0135731 26270047 10.1371/journal.pone.0135731 PMC4535773

[CR72] Swinbanks DD (1982) Intertidal exposure zones: a way to subdivide the shore. J Exp Mar Biol Ecol 62:69–86

[CR73] Tao W, Niu L, Liu F, Cai H, Ou S, Zeng D, Lou Q, Yang Q (2020) Influence of river-tide dynamics on phytoplankton variability and their ecological implications in two Chinese tropical estuaries. Ecol Ind 115:106458

[CR74] URycki DR, Good SP, Crump BC, Chadwick J, Jones GD (2020) River microbiome composition reflects macroscale climatic and geomorphic differences in headwater streams. Front Water 2:574728

[CR75] van der Stel A-X, Wösten MMSM (2019) Regulation of respiratory pathways in campylobacterota: a review. Front Microbiol 10:1719 31417516 10.3389/fmicb.2019.01719 PMC6682613

[CR77] Ward T, Larson J, Meulemans J, Hillmann B, Lynch J, Sidiropoulos D, Spear JR, Caporaso G, Blekhman R, Knight R, Fink R, Knights D (2017) Bugbase predicts organism level microbiome phenotypes. BioRxiv

[CR78] Yeoh YK, Dennis PG, Paungfoo-Lonhienne C, Weber L, Brackin R, Ragan MA, Schmidt S, Hugenholtz, (2017) Evolutionary conservation of a core root microbiome across plant phyla along a tropical soil chronosequence. Nat Commun 8:215 28790312 10.1038/s41467-017-00262-8 PMC5548757

