# Supplementary material for: Tidal levels significantly change bacterial community composition in a tropical estuary during the dry season
Source: Mar Life Sci Technol. 2024 Oct 8;7(1):144–56. doi: 10.1007/s42995-024-00254-w (PMC11871172; doi:10.1007/s42995-024-00254-w)

**Supplementary material**

**Tidal levels change significantly bacterial community composition in a tropical estuary during the dry season**

**Pablo Aguilar^a,b,c,d^, Chantima Piyapong,^e,f^*, Nitcha Chamroensaksri^g^, Pachoenchoke Jintasaeranee^f,h^, and Ruben Sommaruga^a^**

^a^ Department of Ecology, University of Innsbruck, Austria.

^b^ Microbial Complexity Laboratory, Instituto Antofagasta and Centre for Bioengineering and Biotechnology (CeBiB), University of Antofagasta, Chile.

^c^ Department of Biotechnology, Faculty of Marine Sciences and Biological Resources, University of Antofagasta, Chile.

^d^ Millennium Nucleus of Austral Invasive Salmonids - INVASAL, Concepción, Chile.

^e^ Department of Biology, Faculty of Science, Burapha University, Chonburi, 20131, Thailand.

^f^ Center of Excellence on Environmental Health and Toxicology (EHT), OPS, Ministry of Higher Education, Science, Research and Innovation (MHESI), Bangkok, 10400, Thailand.

^g^ National Biobank of Thailand (NBT), National Center for Genetic Engineering and Biotechnology (BIOTEC), National Science and Technology Development Agency (NSTDA), Pathum Thani, 12120, Thailand.

^h^ Department of Aquatic Science, Faculty of Science, Burapha University, Chonburi, 20131, Thailand.

*Correspondence: Chantima Piyapong, Department of Biology, Faculty of Science, Burapha University, Chonburi, 20131, Thailand. chantimap@buu.ac.th

Running title: Bacterial community composition of a tropical estuary

**Supplementary Table 1** Physical parameters measured *in situ* at each sampling point.

**Supplementary Table 2** Significance of environmental parameters in NDMS Analysis

**Supplementary Figure 1** Tidal levels measured at different time points, differentiating between dry (A) and wet (B) seasons. Four tidal levels are detected: HHW (higher high water), LHW (lower low water), HLW (higher low water), and LLW (lower low water).

**
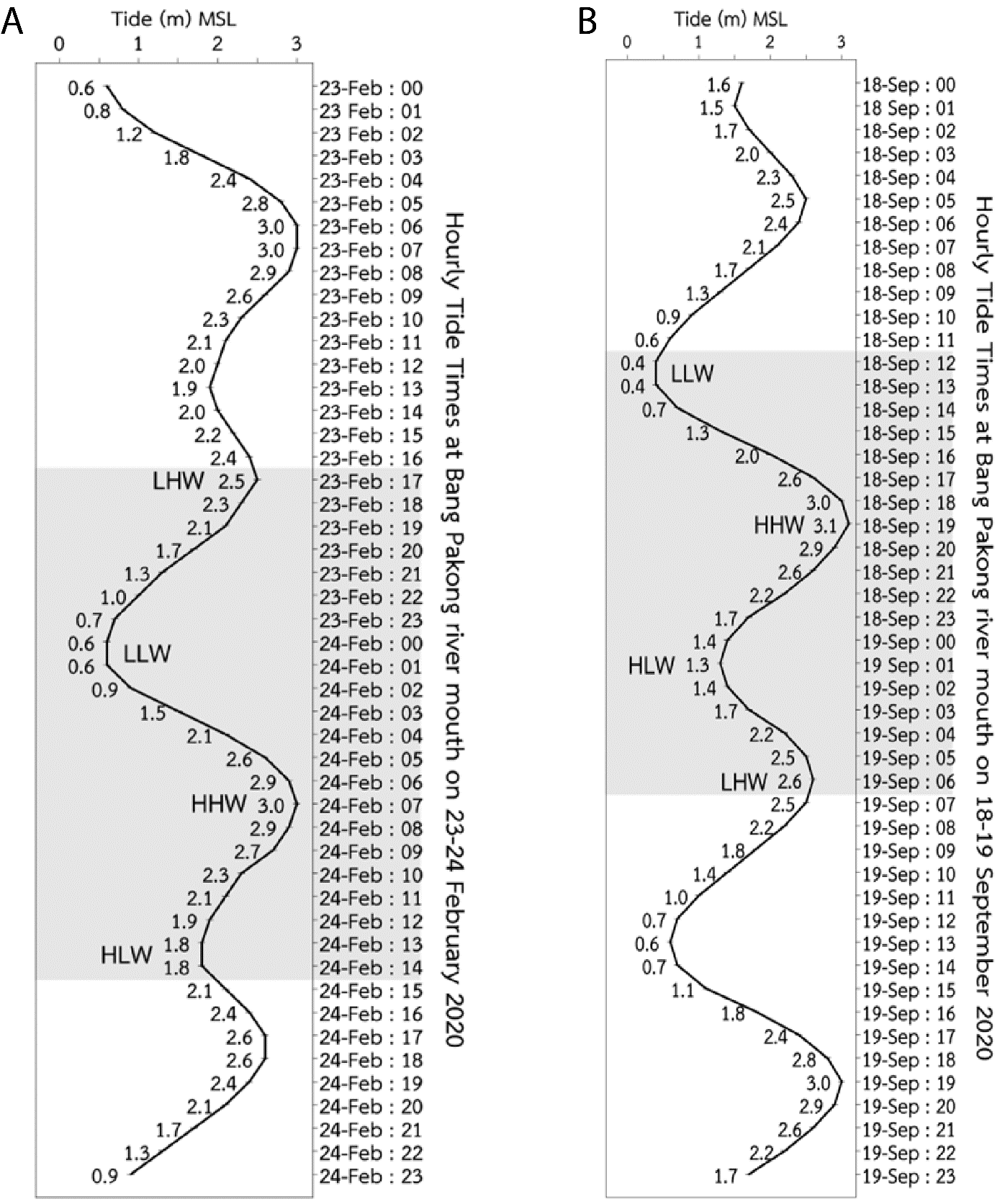
**

**Supplementary Figure 2** Cladogram visualizing the output of the LEfSe algorithm, which identifies significant taxonomical differences between tidal levels within each season. HHW: higher high water. LHW: lower low water. HLW: higher low water. LLW: lower low water.


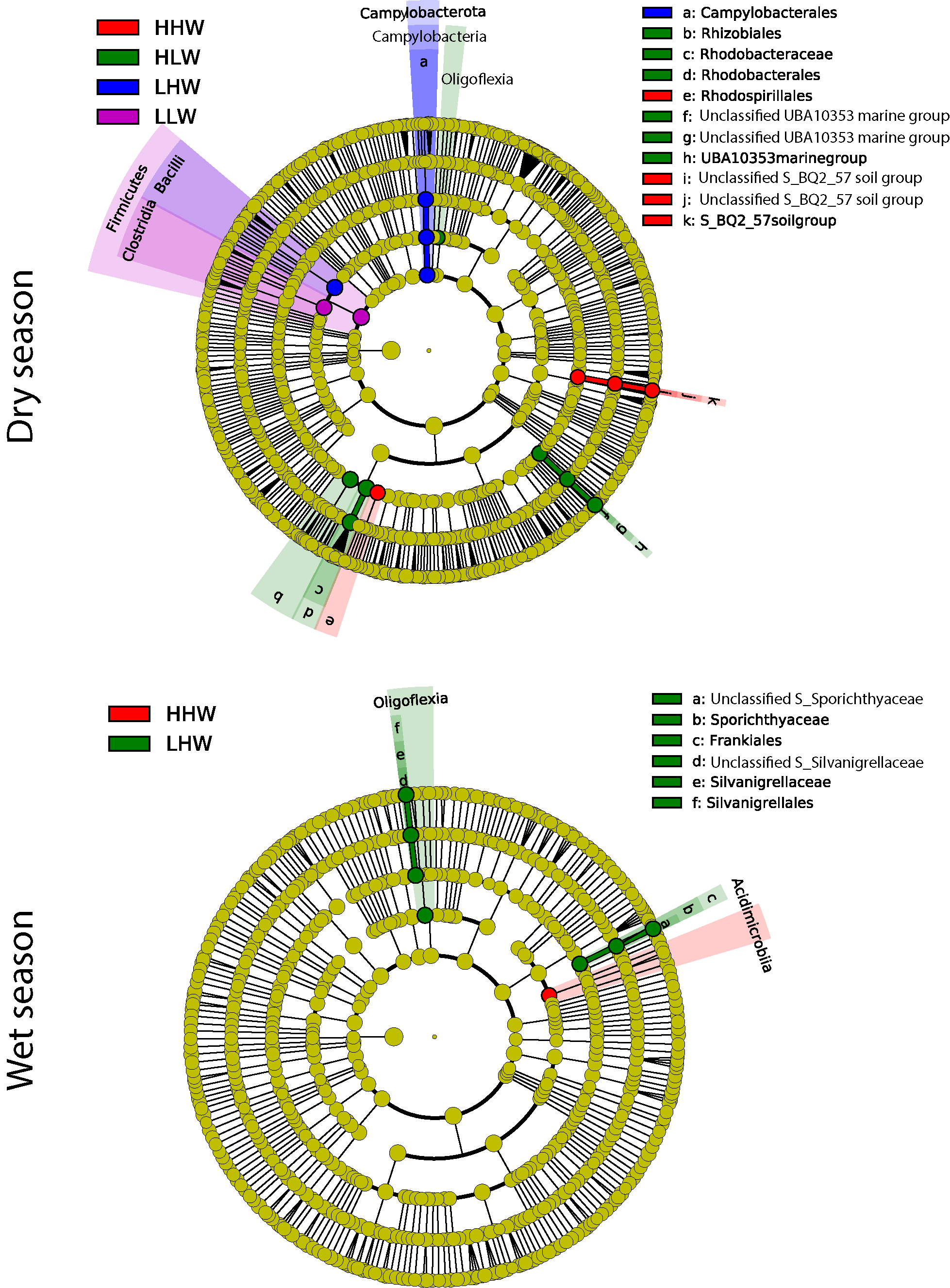


**Supplementary Figure 3** Sampling site at the Bangpakong River, Thailand.


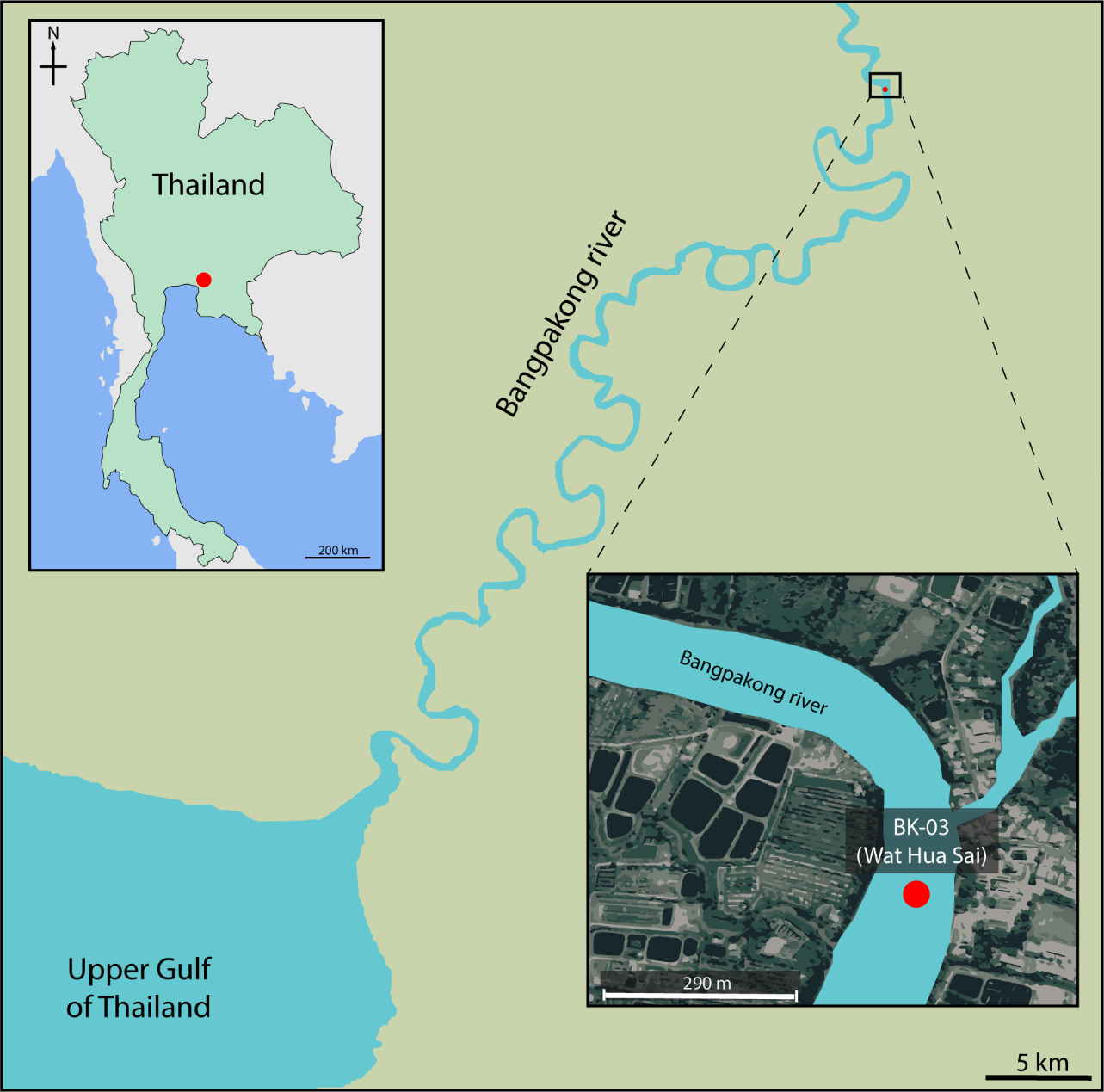

Supplement: Supplementary file 1 — Supplementary file1 (DOCX 929 KB) [file 42995_2024_254_MOESM1_ESM.docx]
